# Germline *CDH1* G212E Missense Variant: Combining Clinical, In Vitro and In Vivo Strategies to Unravel Disease Burden

**DOI:** 10.3390/cancers13174359

**Published:** 2021-08-28

**Authors:** Joana Figueiredo, Fátima Mercadillo, Soraia Melo, Alicia Barroso, Margarida Gonçalves, José Díaz-Tasende, Patrícia Carneiro, Luis Robles, Francisco Colina, Carolina Ibarrola, José Perea, Eurico Morais-de-Sá, Raquel Seruca, Miguel Urioste

**Affiliations:** 1 i3S—Instituto de Investigação e Inovação em Saúde, University of Porto, 4200-135 Porto, Portugal; soraiam@ipatimup.pt (S.M.); m.goncalves@i3s.up.pt (M.G.); pcarneiro@ipatimup.pt (P.C.); eurico.sa@ibmc.up.pt (E.M.-d.-S.); rseruca@ipatimup.pt (R.S.); 2 Institute of Molecular Pathology and Immunology of the University of Porto (IPATIMUP), 4200-135 Porto, Portugal; 3 Familial Cancer Clinical Unit, Spanish National Cancer Research Centre (CNIO), 28029 Madrid, Spain; fmercadillo@cnio.es (F.M.); abarroso@cnio.es (A.B.); 4 Institute for Molecular and Cell Biology (IBMC), University of Porto, 4200-135 Porto, Portugal; 5 Instituto de Ciências Biomédicas de Abel Salazar (ICBAS), Universidade do Porto, 4050-313 Porto, Portugal; 6 Endoscopy Unit, Gastroenterology Department, 12 de Octubre Universitary Hospital, 28041 Madrid, Spain; jose.diazta@salud.madrid.org; 7 Familial Cancer Unit, Medical Oncology Service, 12 de Octubre Universitary Hospital, 28041 Madrid, Spain; luis.robles@salud.madrid.org; 8 Pathology Department, 12 de Octubre Universitary Hospital, 28041 Madrid, Spain or fcolina.hdoc@salud.madrid.org (F.C.); carolina.ibarrola@salud.madrid.org (C.I.); 9 Surgery Department, Fundación Jiménez Díaz University Hospital, 28040 Madrid, Spain; jose.perea@quironsalud.es; 10 Medical Faculty, University of Porto, 4200-319 Porto, Portugal

**Keywords:** HDGC, E-cadherin, *CDH1* missense variant, functional assays

## Abstract

**Simple Summary:**

Hereditary diffuse gastric cancer (HDGC) is an inherited cancer syndrome associated with *CDH1* germline mutations. The increasing detection of *CDH1* genetic variants due to multigene panel testing poses a serious clinical challenge and urges the development of effective classification strategies. In this study, we describe the identification of the novel *CDH1* G212E variant in a large family strongly affected by diffuse gastric cancer. Through a comprehensive characterization pipeline, we provide evidence of the damaging nature of this genetic alteration, thus impacting patient management and family screening.

**Abstract:**

E-cadherin, encoded by *CDH1*, is an essential molecule for epithelial homeostasis, whose loss or aberrant expression results in disturbed cell–cell adhesion, increased cell invasion and metastasis. Carriers of *CDH1* germline mutations have a high risk of developing diffuse gastric cancer and lobular breast cancer, associated with the cancer syndrome Hereditary Diffuse Gastric Cancer (HDGC). The ubiquitous availability of cancer panels has led to the identification of an increasing amount of “incidental” *CDH1* genetic variants that pose a serious clinical challenge. This has sparked intensive research aiming at an accurate classification of the variants and consequent validation of their clinical relevance. The present study addressed the significance of a novel *CDH1* variant, G212E, identified in an unusually large pedigree displaying strong aggregation of diffuse gastric cancer. We undertook a comprehensive pipeline encompassing family data, in silico predictions, in vitro assays and in vivo strategies, which validated the deleterious phenotype induced by this genetic alteration. In particular, we demonstrated that the G212E variant affects the stability and localization, as well as the adhesive and anti-invasive functions of E-cadherin, triggering epithelial disruption and disorganization. Our findings illustrate the clinical implication of a complementary approach for effective variant categorization and patient management.

## 1. Introduction

The unprecedented genomic revolution and emergent next-generation sequencing (NGS) technologies have revealed an increased number of genetic variants and an ensuing challenge in their pathogenicity classification [1,2]. Indeed, the detection of pathogenic germline variants has tremendous implications for personalized diagnostics, surveillance, and therapeutics. The term “precision oncology” was coined to illustrate individualized cancer care as the next logical step following the completion of the Cancer Genome Project in 2014 [3]. However, no consensus has been reached on the interpretation of germline sequence variants, sparking extensive efforts to develop precise and consistent classification tools and criteria [4,5,6].

The impact of pathogenicity prediction has been particularly critical for the clinical management of Hereditary Diffuse Gastric Cancer (HDGC) [7]. HDGC is an autosomal dominant cancer syndrome linked to *CDH1* (E-cadherin) inactivating germline mutations and characterized by high prevalence of diffuse gastric cancer (DGC) and lobular breast cancer (LBC) [8,9]. The cumulative lifetime gastric cancer risk at 80 years of age in carriers of pathogenic *CDH1* germline variants was described as 70% for males and 56% for females, whereas that of breast cancer was reported as 42% for females [10].

Following the first identification of pathogenic germline variants in the *CDH1* gene associated with early-onset diffuse gastric cancer, the International Gastric Cancer Linkage Consortium (IGCLC) has been updating specific guidelines for *CDH1* genetic screening of patients and families at risk [8,11]. To date, more than 155 *CDH1* variants have been described, among which 20% are of the missense type [10]. Of note, despite some scarce evidence, no definite genotype–phenotype correlations have been established based on mutation type or affected protein domain [12].

Upon confirmation of a pathogenic variant, carriers are counselled to undergo prophylactic total gastrectomy, which remains the cornerstone of gastric cancer risk management [11]. Strikingly, histologic examination of resected stomachs of asymptomatic mutation carriers often reveals multiple foci of intramucosal (pT1a) signet ring cell carcinoma (SRCC) [13,14,15]. It has been long debated to what extent do these SRCCs progress to advanced stages or remain indolent in the gastric mucosa. Given this context, and the lack of effective surveillance modalities for carriers, the assessment of the phenotypic impact of *CDH1* variants represents a massive clinical issue. The past decades have witnessed significant improvements in variant interpretation and management of germline carriers [16,17,18,19,20,21,22]. Accordingly, *CDH1* specifications for variant curation guidelines proposed by the American College of Medical Genetics and Genomics, and the Association for Molecular Pathology (ACMG/AMP) have been developed and validated through a systematic evaluation of a large cohort of clinical laboratory data [4,23]. Nonetheless, the majority of these specifications remain inapplicable in missense alterations. Thus, a major goal in HDGC is to establish functional and analytical assays to predict the clinical relevance of *CDH1* missense variants while addressing the biological mechanisms that underlie the aggressive behavior of E-cadherin dysfunctional cells [24].

In this study, we describe an extended family pedigree harboring a *CDH1* missense variant that strongly segregates with diffuse gastric cancer. Through a multidisciplinary pipeline, we provide evidence of the deleterious effects caused by the G212E variant. A notable point of the present work was the comprehensive analysis of the family clinical presentation and meticulous in vitro and in vivo functional characterization of the variant, which combined had a major impact in the clinical management of the proband and extended family members.

## 2. Materials and Methods

### 2.1. Subjects

A Spanish family fulfilling HDGC clinical criteria underwent *CDH1* genetic testing in the Familial Cancer Clinical Unit of the Spanish National Cancer Research Center (CNIO, Madrid, Spain). Individuals enrolled in this study received genetic counselling, in which they were informed about risks of gastric and breast cancer, surveillance options and risk-reducing surgery. Informed consent was obtained from all family members and the study was approved by The Committee for Ethical Research of the Hospital Universitario de Fuenlabrada (Madrid, Spain).

### 2.2. DNA Extraction and Variant Detection

Genomic DNA was extracted from peripheral blood leukocytes using the Maxwell^®^ RSC automatic extractor (Promega, Madison, Wisconsin, WI, USA), following manufacturer’s instructions. Analysis of asymptomatic family relatives was performed based upon recognition of variant segregation with disease and functional evaluation of the variant. In the case of deceased individuals, genomic DNA was obtained from paraffin-embedded normal tissue using the Qiagen DNeasy Blood & Tissue kit (Qiagen, Hilden, Germany). Mutation analysis was achieved through PCR amplification and direct Sanger sequencing of all exons as well as intron–exon boundaries of the *CDH1* gene. PCR conditions and primers can be provided upon request. Bi-directional sequencing was carried out using BigDye Terminator v3.1 Cycle Sequencing Kit (Thermo Fisher Scientific, Waltham, MA, USA) and analyzed on an ABI 3730XL DNA analyzer (Applied Biosystems, Waltham, MA, USA). Resulting sequences were compared with that of reference DNA NG_008021.1. As a control, *CDH1* variant screening was performed by high-performance denaturing liquid chromatography (DHPLC) in a group of seven hundred and thirty-three healthy unrelated individuals of Spanish origin. Variant nomenclature follows the Human Genome Variation Society (HGVS) guidelines.

### 2.3. In Silico Predictions

SIFT (Sorting Intolerant from Tolerant, http://sift.jcvi.org/ accessed on 11 July 2021), PolyPhen-2 (http://genetics.bwh.harvard.edu/pph2/ accessed on 11 July 2021) and FoldX (http://foldxsuite.crg.eu/ accessed on 11 July 2021) algorithms were applied to predict the impact of the variant on protein function [19,25,26]. The SIFT and Polyphen-2 (version 2.2.8) software were run with the Ensembl transcript 261769, substitution G212E. To calculate structural impact, FoldX (version 5) implemented in a Linux environment (Ubuntu 20.04.2 LTS) was used [19,27]. The mutant was induced in the humanized extracellular model of *Xenopus laevis* (PDB 1L3W), using the command Mutate residue. The native-state stability change between mutant and wild-type structures (ΔΔG = ΔGMut − ΔGWT) was automatically generated in 5 runs. Mutations with ΔΔG > 0.8 kcal/mol are considered destabilizing [19].

### 2.4. Plasmids

The E-cadherin variant G212E (c.635G > A) was constructed by site-directed mutagenesis in the h*CDH1* pIRES2-EGFP vector (Clontech, Takara Bio, Shiga, Japan), following the protocol described by Wang and Wilkinson [28]. The corresponding empty vector (Mock) was used as control. All cloning vectors were verified by direct sequencing.

### 2.5. Cell Culture and Transfection

CHO cells (Chinese Hamster Ovary, ATCC number: CCL-61) were grown at 37 °C under 5% CO_2_ humidified air, and cultured in α-MEM medium (Gibco, Invitrogen, Waltham, MA, USA) supplemented with 10% fetal bovine serum (HyClone, Perbio) and 1% penicillin/streptomycin (Gibco, Invitrogen, Waltham, MA, USA). For transfection, 1.6 × 10^5^ cells were seeded in 6-well plates and, 24 h later, cells were transiently transfected with vectors encoding either the wild-type protein, the variant G212E or the empty vector (Mock condition). Transfection was performed using Lipofectamine 2000 (Invitrogen, Waltham, MA, USA) according to manufacturer’s recommendations. Transfection efficiency was controlled in each experiment by flow cytometry, measuring GFP fluorescence.

### 2.6. Western Blotting

Cell lysates were prepared by cell scraping in cold Catenin Buffer, whose composition is 1% Triton X-100 (Sigma-Aldrich, St. Louis, MI, USA) and 1% Nonidet P-40 (Sigma-Aldrich, St. Louis, MI, USA) in PBS, supplemented with a cocktail of phosphatase (Sigma-Aldrich, St. Louis, MI, USA) and protease inhibitors (Roche, Basel, Switzerland). Protein concentration of cellular extracts was determined using a modified Bradford assay (Bio-Rad, Hercules, CA, USA). For analysis, 15 μg of total protein were diluted in 4× Laemmli buffer (Bio-Rad) with β-mercaptoethanol (Sigma-Aldrich, St. Louis, MI, USA), separated in 7.5% SDS–PAGE gels and electroblotted onto Hybond ECL membranes (Amersham Biosciences, Amersham, UK). Membranes were blocked in 5% non-fat milk and 0.5% Tween-20 in PBS for 1 h, and immunoblotted with antibodies against E-cadherin (1:2500, Clone HECD1, Invitrogen, Waltham, MA, USA) and α-Tubulin (1:10000, Sigma-Aldrich, St. Louis, MI, USA). The secondary antibody sheep anti-mouse HRP-conjugated (Amersham Biosciences, Amersham, UK) was used, followed by detection with ECL reagents (Amersham Biosciences, Amersham, UK). Immunoblots were quantified using Quantity One Software (Bio-Rad, Hercules, California, CA, USA). The whole western blot figures can be found in the supplementary files. 

### 2.7. Immunofluorescence Staining and Expression Profiling

Cells seeded on top of glass coverslips were washed in PBS, fixed on ice-cold methanol for 20 min and blocked with 3% BSA in PBS for 30 min, at room temperature. E-cadherin was stained with a specific mouse monoclonal antibody (BD Biosciences, Franklin Lakes, NJ, USA), diluted at 1:300 in blocking solution. The Alexa Fluor 488 goat anti-mouse (1:500, Invitrogen, Waltham, MA, USA) was applied as secondary antibody for 1 h in the dark. Coverslips were mounted on slides using Vectashield with DAPI (Vector Laboratories, Burlingame, CA, USA). Images were acquired on a Carl Zeiss Apotome Axiovert 200 M Fluorescence Microscope with an Axiocam HRm camera, and processed with Zeiss Axion Vision 4.8 software. For quantitative purposes, the intensity of fluorescent signals that occur between two contiguous cells (nuclei) was extracted as described by Sanches et al. [21,29]. Position 1 corresponds to the geometric center of nucleus 1, position 100 is the center of nucleus 2, whereas position 50 represents the plasma membrane. Signal intensity of each position from 1 to 100 was obtained and statistically examined.

### 2.8. Matrigel Invasion Assay

Cell invasion was assessed with Matrigel coated chambers suitable for 24-well-plates (Corning Biocoat). Matrigel inserts were hydrated by filling the inner and outer compartments with α-MEM medium for 1 h at 37 °C. Thereafter, 500 µL of a cellular suspension at 5 × 10^4^ cells/mL (corresponding to 2.5 × 10^4^ cells) were plated in each insert, and the plate was incubated at 37 °C in a humidified atmosphere with 5% CO_2_. Following the 24 h seeding, a pre-wet ‘cotton swab’ was used to remove non-invasive cells and Matrigel from the upper side of the filters. The filters were then washed in PBS, fixed on ice-cold methanol for 15 min, and mounted on Vectashield with DAPI (Vector Laboratories, Burlingame, CA, USA). The total number of invasive nuclei present in the bottom of each filter was counted under a Leica DM2000 microscope.

### 2.9. Slow Aggregation Assay

Cell–cell aggregation was analyzed in 96-well-plates coated with 50 µL of an agar solution, prepared by solving 100 mg of Bacto-Agar in 15 mL of sterile PBS [16,20]. A cellular suspension of 1 × 10^5^ cells/mL was prepared for each condition, and 200 µL (corresponding to 2 × 10^4^ cells) were added to the agar-coated wells. Triplicates were used to validate experimental results. The plate was then incubated at 37 °C under 5% CO_2_ humidified air. Aggregation phenotypes were observed and photographed 24 h and 48 h after seeding, under a Leica DMi1 inverted microscope with camera. Cellular aggregates were quantified by assessing their area in Fiji [30].

### 2.10. Cell Network Analysis

Cellular networks were generated based upon nuclei geometric centers computed from images of DAPI-stained cells. Denoising and nuclei segmentation were performed in each image by applying the Otsu method and the Moore–Neighbor tracing algorithm, modified by Jacob’s stopping criteria, as previously described [22]. Nuclei geometric centers were then calculated and connected using the Delaunay triangulation algorithm [31]. Geometric features of triangles composing the generated networks were explored with the MatLab tool.

### 2.11. Generation of Drosophila Stocks

UAS-driven constructs to express human *CDH1* were created using the Gateway Cloning System (Life Technologies, Carlsbad, CA, USA). Site-directed mutagenesis (c.635G > A) was performed to generate pENTR-*CDH1*(G212E) using the pENTR-*CDH1* vector template. A new gateway destination vector, pPW-attB, was produced to enable PhiC31 site-specific insertion of UAS-driven transgenes encoding untagged proteins. With this purpose, the pPMW-attB (gift from Frederique Peronnet, Addgene plasmid # 61814) was digested with NsiI (New England BioLabs Inc., Ipswich, Massachusetts, USA) to subsequently subclone a fragment containing the attB site into pPW (Gateway library). Final constructs were obtained using LR clonase II-mediated recombination of pENTR-*CDH1* and pENTR-*CDH1*(G212E) with pPW-attB. UAS-*CDH1* and UAS-*CDH1*(G212E) transgenes were then inserted into the attP40 landing site via PhiC31 site-specific transgenesis (BestGene Inc, Chino Hills, CA, USA), placing wild-type and mutated cadherin under the same genetic environment.

### 2.12. Drosophila Genetics

Clonal analysis using the FLPout system [32] was used to evaluate the impact of *CDH1* variant expression in the Drosophila follicular epithelium. This enabled direct comparison between expressing and non-expressing clones within mosaic egg chambers. Briefly, UAS-*CDH1* transgenic lines were crossed with y w hsFlp; tub-FRT-stop-FRT-Gal4, UAS-GFP/CyO. The progeny (y w hsFlp/+; UAS-*CDH1*/ tub-FRT-stop-FRT-Gal4, UAS:GFP) was heat-shocked at 37 °C to randomly induce Flippase-mediated removal of the FRT cassette, and subsequent expression of GAL4/UAS-driven human cadherin.

### 2.13. Ovary Immunofluorescence and Imaging

Drosophila ovaries were dissected in Schneider’s Insect Medium (Sigma-Aldrich, St. Louis, MI, USA) supplemented with 10% FBS. Fixation was performed in 4% paraformaldehyde for 20 min, followed by washing steps with 0.05% Tween-20 in PBS, and blocking with 10% BSA in PBS-T. Primary antibodies were applied overnight (mouse anti-E-cadherin, 1:500, Invitrogen, Waltham, Massachusetts, USA; rabbit anti-aPKC, 1:250, Santa Cruz Biotech, Dallas, TX, USA). After washing steps in PBS-T supplemented with 1% BSA, ovaries were incubated for 2 h in the dark with secondary antibodies (Alexa Fluor 561 goat anti-mouse, 1:300, or the Alexa Fluor 647 goat anti-rabbit, 1:100, Invitrogen, Waltham, MA, USA). Actin structures were stained using phalloidin Cruzfluor 647 conjugate (Santa Cruz Biotech, Dallas, TX, USA). Ovaries were mounted on Vectashield with DAPI (Vector Laboratories, Burlingame, CA, USA) and imaged using an inverted laser scanning confocal microscope (Leica TCS SP5 II, Leica Microsystems, Wetzlar, Germany). Image processing was achieved using Leica Application Suite software (LAS version 2.6).

### 2.14. Statistical Analysis

Data were statistically analyzed using the two-tailed unpaired or paired Student’s *t*-test as indicated. The Wilcoxon signed-rank test was applied to internuclear profile data. All analyses were performed using the GraphPad Prism software (version 7.04) and *p* ≤ 0.05 was required for statistical significance.

## 3. Results

### 3.1. The G212E E-Cadherin Variant Segregates with Diffuse Gastric Cancer within a Large Family Pedigree

**Figure 1:**
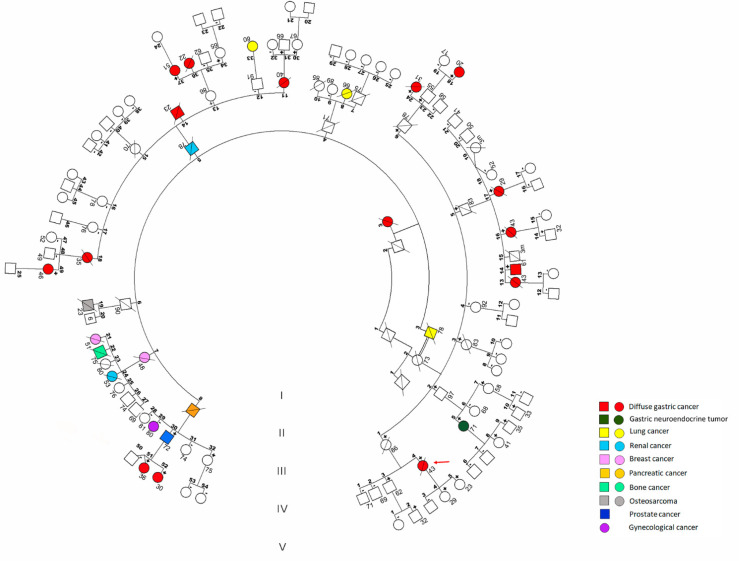
Clinical presentation associated with the G212E E-cadherin variant. Pedigree illustrating variant segregation and disease phenotypes within a family structure along five generations (I-V). For simplicity, individuals were numbered in each generation. Squares indicate males and circles indicate females. Symbols with a slash denote deceased individuals. Symbols + or − in the upper right corner represent the result of *CDH1* variant screening. The proband is identified with a red arrow. Cancer types affecting family members are specified by color. The actual age (May 2021) or the age at the time of death is displayed below each individual. m stands for months.

In this work, we describe an unusually large family with strong aggregation of diffuse gastric cancer (Figure 1). The proband was identified in 2005 as a 41-year-old woman diagnosed with DGC with signet ring cells one year prior to consultation (Subject IV-4). She described the family with numerous consanguineous marriages (only partially shown in the pedigree) and reported four first cousins affected by DGC: Subject IV-24, 30 years old, diagnosed two years earlier; and three sisters (Subjects IV-13, IV-16 and IV-17) who died at the age of 43, 43 and 29, respectively. In addition, four other cases of DGC had occurred in relatives of third or fourth degree, including the maternal great grandmother (Subject I-3); Subject III-11, who died at the age of 40; and her siblings (Subjects III-14 and III-18), who died at the age of 23 and 35, respectively. An additional case appeared in a fifth-degree relative (Subject IV-36) who died at the age of 22. A panel of other cancer types were also described, namely two cases of breast cancer of unspecified type in a mother and daughter (Subject II-7 and Subject III-21); and a gastric neuroendocrine tumor (Subject IV-5), among others. Neither patients with cleft lip/cleft palate, or with other birth defects, nor couples with a history of recurrent miscarriages were identified.

Following HDGC genetic testing criteria, *CDH1* screening was offered to the proband, which identified the missense variant c.635G > A (p.G212E) in exon 5 of the gene. The *CDH1* gene was also screened in Subject IV-24, another living relative affected by DGC at the time. In parallel, we have analyzed microsatellite instability and the expression of mismatch repair proteins in a paraffin tumor sample from Subject IV-24, which was shown to be a stable tumor with intact MLH1, MSH2, MSH6, and PMS2 proteins. The study was extended to deceased relatives (Subjects IV-16 and IV-17) diagnosed with DGC. All turned out to be carriers of the G212E variant. Upon genetic counselling, evaluation of *CDH1* status was offered to other family members.

In total, we have analyzed 83 family members, 32 of which were carriers of the mutation. All living carriers have been advised to consider prophylactic total gastrectomy (PTG), but only two individuals have opted to do so. Regarding histopathological analysis, we were able to collect information from the gastrectomy specimen of Subject IV-23, yielding a macroscopically and microscopically normal stomach. The remaining family members declined or chose to postpone PTG. Importantly, all of them undergo an annual endoscopy with multiple biopsies throughout different anatomical zones of the stomach, carried out by endoscopists with solid experience in HDGC management. Breast surveillance in female carriers is pursued by alternating mammography and MRI with 6-month intervals.

Endoscopic surveillance allowed the identification of grossly visible tumors in individuals IV-49 and V-18, and tiny foci of SRCC in subjects IV-14, 37, 51, and 52, who then underwent total gastrectomy.

### 3.2. Histopathogical Findings Are Compatible with HDGC Clinical Presentation

Histopathological evaluation of the cases first identified in the family (Subjects IV-4, 13,16, 17, 24) showed extensive, diffuse, poorly differentiated adenocarcinomas with SRC component eroding the mucosa and invading throughout the lamina propria, muscularis mucosae, submucosa and muscularis propria, further penetrating the subserosal connective tissue with invasion of the peritoneum and metastases in the regional lymph nodes.

In contrast, cases diagnosed by follow-up endoscopic examination presented less advanced lesions. Among the patients who had a positive biopsy for carcinoma during endoscopic surveillance and underwent total gastrectomy, only one had a grossly visible ulcerated tumor in the cardia (1.8 cm, Subject IV-49). In the remaining cases, no tumors were detected by endoscopy and three cases were found positive for carcinoma in the 2nd, 3rd (6 months to 1 year) and 23rd (13 years) screening biopsies (Table 1).

**Figure 2:**
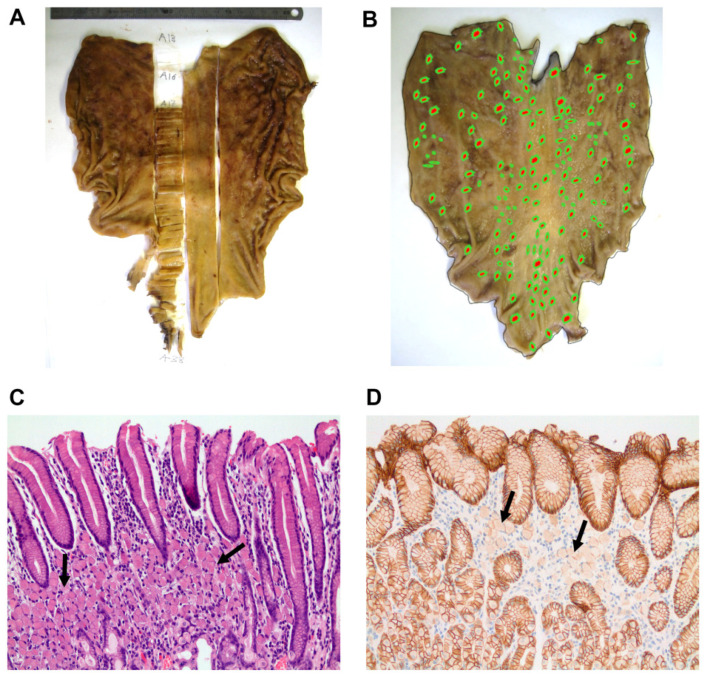
Macroscopic and microscopic features of gastrectomy specimens. (**A**) Procedure used to include entire stomachs for systematic histological analysis. (**B**) Gastrectomy specimen from Subject IV-51. Dots indicate the sites where microscopic lesions were identified. (**C**) Representative image of a focus of signed ring cell carcinoma restricted to the upper half of the mucosa (Subject IV-52, hematoxilin-eosin staining, ×200 magnification, arrows). (**D**) Immunohistochemistry showing intact membrane expression of E-cadherin in the non-tumoral foveolar epithelium and focal loss of staining in tumor cells from Subject IV-52 (×200 magnification, arrows).

Macroscopically, most gastrectomy specimens appeared normal to the naked eye and complete stomachs were thus included for histopathological examination with topographical location recorded for each sample (Figure 2A). At the microscopic level, all cases displayed multiple intramucosal foci (less than 8 mm in diameter) of diffuse, poorly cohesive signet ring cell carcinoma of classic morphology. The foci were randomly distributed across the different regions of the stomach in all cases (Figure 2B), although in two cases they were more predominant in the lesser curvature and body area. Furthermore, there was a large variation in the number of foci detected, ranging from 9 to 169, and no relationship was found between their number and patient’s age (Table 1). With the exception of Subject IV-49, who exhibited tumor cells extending into the muscularis propria (T2), the other cases displayed signet ring cells restricted to the upper half of the lamina propria (T1a, Figure 2C). No lymphovascular or perineural invasion was observed in any case and no regional lymph node metastases were detected in the lymphadenectomies. Remarkably, immunohistochemical studies demonstrated typical loss of membranous E-cadherin staining in all cases analyzed (Figure 2D).

More recently, an additional case was diagnosed in a first endoscopic surveillance examination of a 19-year-old subject (Subject V-18). Following the detection of a thickening in the central region of the body of the stomach, a biopsy identified a poorly cohesive adenocarcinoma. The patient received neoadjuvant chemotherapy, followed by total gastrectomy that revealed a yT2N0 DGC invading the muscularis propria without regional lymph node metastases.

### 3.3. In Silico Analysis Predicts Destabilization of E-Cadherin Structure by the G212E Variant

Following the identification of the variant in the proband and in several close relatives affected by DGC, we pursued a framework aiming to determine the significance of the G212E variant. Although this variant has been reported in LOVD database (https://databases.lovd.nl/ accessed on 2 July 2021), it was not submitted to HDMD (http://www.hgmd.cf.ac.uk/ac/index.php/ accessed on 2 July 2021) or ClinVar (https://www.ncbi.nlm.nih.gov/clinvar/ accessed on 2 July 2021) registries. Likewise, there were no data on variant frequency in large genomic databases, such as The Genome Aggregation Database (gnomAD; https://gnomad.broadinstitute.org/ accessed on 2 July 2021), arguing in favor of a rare and potentially deleterious alteration. In addition, we evaluated its presence in a series of 733 healthy controls of the Spanish population through DHPLC and verified that this alteration was absent from this cohort.

To access the pathogenicity of the G212E variant, we next performed in silico analysis through SIFT, PolyPhen-2 and FoldX software, thus considering not only sequence homology, but also structural information and physical properties of amino acids [19,25,26]. Of note, all bioinformatic tools were consistent and predicted that this variant affects the function of E-cadherin. G212E substitution was considered “Damaging” by SIFT with a score of 0.008 (scores bellow 0.05 are considered to be Damaging); PolyPhen-2 classified the variant as “Probably Damaging” with the highest possible score (score 1.0); and FoldX modelling pinpointed a highly destabilized protein structure, with a striking energetic difference between the mutant and the WT reference of 14.98 kcal/mol (mutations associated to structural impact present differences >0.8 kcal/mol) [19].

### 3.4. The CDH1 G212E Variant Yields Abnormal E-Cadherin Levels and Distribution Profiles

**Figure 3:**
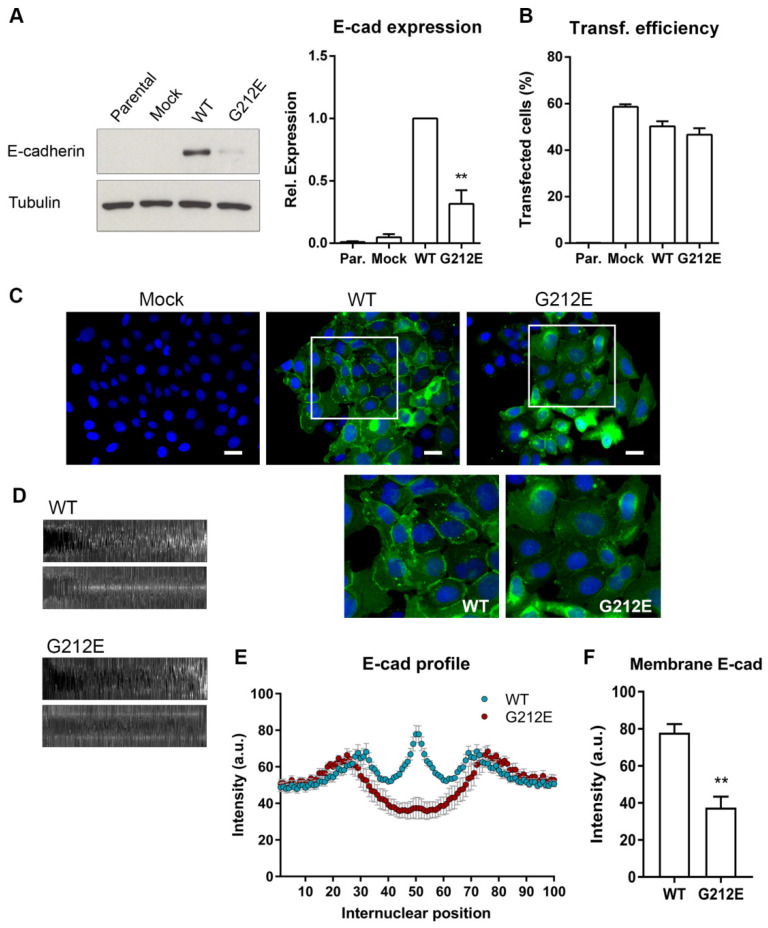
Expression profile of the E-cadherin missense variant G212E. (**A**) E-cadherin protein expression was analyzed by Western Blot in CHO cells transfected with wild-type E-cadherin, the variant G212E, and the empty vector (Mock). α-Tubulin was used as loading control. The intensity of the bands was quantified and normalized against wild-type E-cadherin cells. In the graph, bars represent average + SE of E-cadherin protein levels in four independent experiments. (**B**) Transfection efficiency was evaluated by flow cytometry using GFP levels. (**C**) Immunofluorescence showing E-cadherin localization (green staining) of wild-type, G212E, and Mock cells. Nuclei were counterstained with DAPI (blue). Scale bars represent 20 µm. Enlarged image showing the diffuse pattern of G212E E-cadherin, when compared with that of the wild-type protein at the plasma membrane. (**D**) Internuclear profiles encompassing signal intensities along contiguous cells were extracted (upper map) and geometrically compensated (bottom map). (**E**) Quantification of mutant and wild-type E-cadherin expression profiles. (**F**) Graph displays mean + SE of fluorescence intensity at the internuclear position 50, which corresponds to the plasma membrane. ** represents *p* ≤ 0.01.

To test the impact of the mutant protein in vitro, we transiently transfected CHO cells with vectors encoding wild-type E-cadherin, the G212E variant and the empty vector, as a control. CHO cells were chosen given that they are completely negative for E-cadherin expression and constitute the most well-established model for studying the pathogenic relevance of missense mutations [33,34,35]. By analyzing total protein levels, we verified that mutant cells present significantly decreased E-cadherin expression when compared with those expressing wild-type protein (from 1.0 to 0.32-fold, *p* = 0.0083), despite similar transfection efficiencies (Figure 3A,B). This is consistent with protein destabilization and premature degradation, which supports the in silico results and may reflect the post-translational regulation mechanisms previously described for missense mutants [19,20,36].

Immunofluorescence showed that G212E cells display a diffuse pattern of E-cadherin throughout the cytoplasm and no protein enrichment at the plasma membrane, in contrast to wild-type expressing cells, which present a strong staining at the membrane (Figure 3C). For quantitative assessment of protein localization, we have applied a bioimaging approach that captures and compiles fluorescence signals between contiguous cells [29]. As observed in Figure 3D, the wild-type map exhibits significantly more intense E-cadherin when compared with the G212E map (*p* = 0.0005). More so, the profile of wild-type cells is characterized by a maximum intensity peak in the region that represents the plasma membrane (position 50). Contrarily, cells expressing the G212E variant show much weaker pixel intensity at the same position, with maximum intensity levels observed at a specific position in proximity to each nucleus (positions 25 and 76, Figure 3E,F), compatible with a possible accumulation in the perinuclear endoplasmic reticulum.

### 3.5. The G212E Variant Compromises Protein Function and Cell-Cell Adhesion

**Figure 4:**
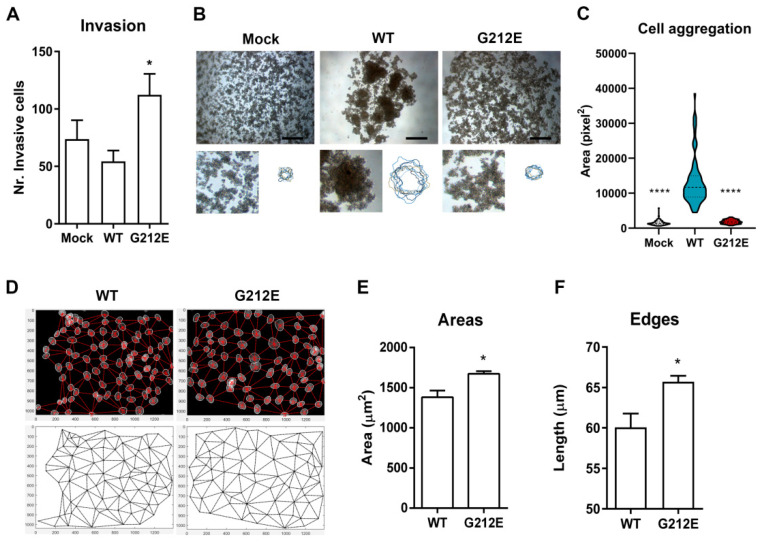
In vitro functional effects elicited by the E-cadherin missense variant G212E. (**A**) Invasive ability mean + SE of cells transfected with the empty vector, as well as with wild-type and G212E E-cadherin forms. (**B**) Aggregation phenotypes of the different cell lines. Scale bars represent 400 µm. Image insights show cellular structures in detail. Scheme illustrating the outlines of 10 aggregates formed by wild-type or mutant cells. (**C**) Quantification of aggregate area. (**D**) Computational analysis of cellular distribution patterns based upon networks of neighboring nuclei. Quantitative features of networks, area (**E**) and edges length (**F**). * represents p ≤0.05 and **** p ≤0.0001.

To investigate the functional significance of the G212E variant, we next explored Matrigel invasion assays and slow aggregation experiments. We verified that the variant induces an increase in the number of cells that are able to invade a matrix (54.3 wild-type cells versus 112.3 mutant cells, *p* = 0.048, Figure 4A), and has a strong effect in the capacity to mediate homotypical cell–cell adhesions (Figure 4B). While cells expressing wild-type E-cadherin spontaneously aggregate upon seeding on a semisolid substrate, cells expressing the G212E mutant present a scattered phenotype, appearing isolated and homogenously distributed in the agar (Figure 4B). In fact, wild-type cells form large and compact aggregates with an average area of 13,366 pixel^2^, whereas the cellular structures yielded by mutant cells reach on average 1845 pixel^2^ (*p* < 0.0001), similar to those of the Mock control (1506 pixel^2^, Figure 4C).

We then applied an algorithm to address whether the G212E variant generates an abnormal epithelial organization [22]. Networks connecting neighboring nuclei of G212E cells were clearly distinct from those of the wild-type counterparts (Figure 4D). In particular, networks from mutant cells present significantly higher triplet areas (*p* = 0.029) and internuclear distances (edges, *p* = 0.0385) when compared with E-cadherin competent cells (Figure 4E,F), suggesting that mutant cells are loosely attached. We thus conclude that the G212E variant affects the ability of E-cadherin to establish normal cell adhesion and may therefore impact epithelial architecture.

### 3.6. Expression of Human G212E Variant Causes Epithelial Disruption in Drosophila

**Figure 5:**
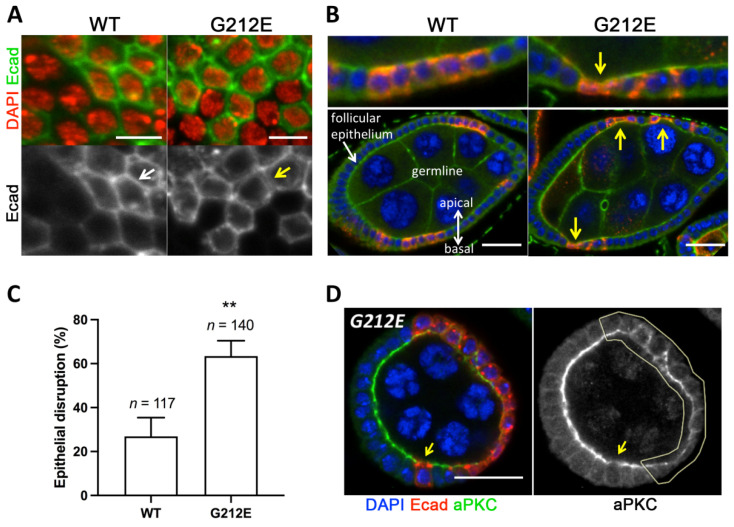
Structural features of human G212E variant in Drosophila epithelia. (**A**) Surface views of the Drosophila follicular epithelium with mosaic clones expressing human wild-type *CDH1* and the G212E variant using the FLPout/tub-GAL4 system. Cells expressing G212E E-cadherin display weaker accumulation at the plasma membrane (arrows). E-cadherin is shown in green, and DAPI (red) labels the nucleus. Scale bars represent 5 µm. (**B**) Longitudinal view of egg chambers stained for E-cadherin (red), actin (green) and DAPI (blue). Clones expressing G212E lead to epithelial invaginations with consequent tissue disruption (arrows). Upper images are close-ups of the lower ones. Scale bars represent 20 µm. (**C**) Quantification of disrupted epithelial architecture caused by the overexpression of wild-type and mutant E-cadherin in the Drosophila follicular epithelium. Data derives from four independent experiments. For each experiment, around 30 clones were quantified per genotype (total number in the graph). Graph displays mean + SE; ** represents *p* ≤ 0.01 for a two-tailed paired t-test. (**D**) Longitudinal section of an egg chamber with a large clone of cells expressing the G212E mutant (red) and stained for the apical marker aPKC (green). Loss of apico-basal polarized localization is evident even in a single cell overexpressing G212E (arrow). Scale bar represents 20 µm.

To further explore the effects of G212E in epithelia, we have established an in vivo model in *Drosophila melanogaster*. For that purpose, human *CDH1* and *CDH1* G212E were expressed in the Drosophila follicular epithelium using the FLPout/tub-GAL4 system [32], which enables ectopic expression of human E-cadherin and direct comparison of epithelial shape within a mosaic tissue. We first studied the expression and localization of human E-cadherin in the surface of egg chambers, and verified that the G212E is weakly expressed at the plasma membrane when compared with the well-defined pattern of the wild-type protein (Figure 5A). Interestingly, we observed that clones expressing the G212E mutant, including those with only two or three expressing cells, induce epithelial invaginations, compromising tissue architecture and integrity (Figure 5B). Accordingly, a large proportion of G212E expressing cells disrupt normal epithelial architecture, an effect rarely observed upon overexpression of *CDH1* wild-type (Figure 5B,C). Lastly, we have assessed whether expression of G212E could affect apical–basal organization using the apical marker aPKC. The apical enrichment of aPKC was found to be decreased in G212E expressing cells when compared with non-expressing tissue (Figure 5D). Thus, expression of G212E may also inhibit apical identity, further corroborating the damaging nature of the variant.

## 4. Discussion

Herein, we describe a large Spanish family fulfilling HDGC criteria and carrying the novel G212E *CDH1* germline missense variant. In this family, 16 of its members, 14 females and 2 males, have developed diffuse gastric carcinoma. Among the affected members, 10 died prior to or during the genetic study process while the other 6 were diagnosed with the disease upon implementation of surveillance measures. Importantly, these latter remain alive and disease free, thus constituting an irrefutable proof of the value of accurate genetic counselling coupled with a comprehensive evaluation of genetic variants.

In the present family, the onset of disease was below 45 years of age, with the exception of Subjects IV-49 and IV-14, who were diagnosed at the age of 46 and 54, respectively. Indeed, 42.3% of affected individuals were diagnosed before the third decade of their life (Subjects III-14, IV-17, 36, 51, and 52) with the youngest patient at 19 years of age (Subject V-18). This age range justifies genetic testing and initiation of a surveillance program in asymptomatic minors. Concerning prophylactic procedures, there was only one case with available information, and upon an exhaustive microscopic screening no pre-invasive lesions were identified. It would have been extremely important to evaluate the additional gastrectomy specimens and compare them to previous reports demonstrating that, in fact, 95.3% of gastrectomies display pre-invasive or early invasive lesions [13].

In accordance with prior studies regarding missense variants, we verified that disease penetrance in this family is lower than that described by Hansford et al. [10,37]. Strikingly, a number of long-lived carriers in the III generation remained disease free. This lower penetrance may be related to residual protein production and function in the mutant context, or with different severity grades possibly dependent on the compromised *CDH1* domains [20,38,39]. Unexpectedly, we found a difference in the sex ratio of patients affected by DGC, with females showing a high proportion of cases (14/69 female versus 2/53 male). We speculate that individual genetic backgrounds and/or lifestyle factors may contribute to this biased proportion.

In the last decade, we and others have provided evidence that the disease spectrum of *CDH1* mutation carriers includes DGC, lobular breast cancer, cleft lip/palate, and the blepharocheilodontic syndrome [40,41,42,43]. Some studies have also reported cases of colorectal carcinoma associated with loss of E-cadherin function, although its inclusion in *CDH1* clinical presentation remains controversial [44,45]. Based on our findings, we cannot infer as to whether G212E variant increases the risk of developing other tumors apart from DGC. The two cases affected by breast cancer were not tested for *CDH1* alterations and their histological subtype is currently not known. Moreover, mutation carriers have developed a neuroendocrine tumor (Subject IV-5) and a prostate cancer (Subject III-30) at advanced ages, suggesting a sporadic occurrence rather than a specific inherited susceptibility.

Given the debate surrounding the relevance of missense variants, we developed a pipeline to evaluate the effects of the G212E variant at cellular and tissue levels, following its identification by screening. Using in silico approaches, we collected data pointing to a deleterious impact of the alteration since it substitutes a highly conserved glycine by a glutamine acid, which is predicted to induce a drastic modification in protein structure. In vitro, we were able to demonstrate that the variant generates abnormal levels and distribution of E-cadherin. Specifically, we detected a significant decrease in protein expression and, consequently, its absence from the plasma membrane where it is normally strongly expressed. A small fraction of the protein is present instead in perinuclear patches or diffusely distributed across the cytoplasm, corroborating the activation of mechanisms of protein quality control, as previously described for this type of mutation [19,36]. Consistent with the lack of membrane enrichment, we showed that the G212E mutant hinders the cell-cell adhesive function of E-cadherin and increases cell motility within a Matrigel matrix mimicking the human basement membrane [46,47]. A looser interaction between G212E expressing cells was also uncovered in comparison with the organized monolayer formed by wild-type counterparts.

The consequences of the G212E variant were further investigated at the tissue level using a Drosophila model engineered to express human E-cadherin in the follicular epithelium, which has been extensively used to study epithelial organization and to address mechanisms relevant for human cancer [48,49]. We found that the G212E variant yielded lower levels of E-cadherin at cell–cell junctions and led to pronounced changes in tissue architecture. By monitoring the apical marker aPKC, we further confirmed loss of apical–basal polarity that has been strongly associated with cancer progression [50]. HDGC in particular has been previously proposed to be a clinical manifestation of loss of cell polarity that possibly arises due to abrogation of the role of E-cadherin in mitotic spindle orientation [51,52]. Cell division asymmetry results in deposition of daughter cells in the lamina propria, which subsequently expand and differentiate into SRCC [51].

## 5. Conclusions

This work validates the damaging signature of a novel E-cadherin missense variant in a large pedigree and highlights the potential of an effective variant classification by combining in vitro and in vivo models. In particular, we demonstrate that the G212E alteration compromises protein stability, cell adhesive and invasive properties, as well as tissue integrity, culminating in a severe cancer phenotype such as that seen in HDGC. Our findings proved to impact management of individuals harboring *CDH1* germline alterations and to be crucial for cancer risk estimation.

## Figures and Tables

**Table 1:** Characteristics of patients diagnosed during follow-up endoscopic examination. For each subject, available information is provided on age at diagnosis, surveillance period, screening biopsies up to gastrectomy, number of paraffin-embedded tissue samples, number and anatomical distribution of detected cancer foci, as well as tumor features.

Subject	Age at Diagnosis	Surveillance Period	Screening Biopsies up to Gastrectomy (nr)	Paraffin-Embedded Tissue Blocks (nr)	Intramucosal Foci(nr and Size)	Foci Anatomical Location	Visible Tumour (nr) TNM Localization Size	Histological Features
**IV-14**	59	13 y	23	315	16 <2 mm	All of the stomach, more frequent in body and fundus	No T1aN0	Superficial half mucosa
**IV-37**	43	6 m	2	208	9 <5 mm	All of the stomach	No T1aN0	Superficial half mucosa
**IV-49**	46	no	1	209	32 <2 mm	All of the stomach, more frequent in lesser curvature	Yes (1) T2N0 Cardia ulcerated 1.8 cm	Superficial half mucosa
**IV-51**	28	1 y	3	214	169 <2 mm	All of the stomach	No T1aN0	Superficial half mucosa
**IV-52**	27	7 m	2	202	54 < 8 mm	All of the stomach, more frequent in lesser curvature and posterior wall	No T1aN0	Superficial half mucosa

## Data Availability

Data presented in this study are available upon reasonable request to the corresponding author.

## Supplementary Materials

The following are available online at https://www.mdpi.com/article/10.3390/cancers13174359/s1, The whole western blot figures.
