# Supplementary material for: Germline CDH1 G212E Missense Variant: Combining Clinical, In Vitro and In Vivo Strategies to Unravel Disease Burden
Source: Cancers (Basel). 2021 Aug 28;13(17):4359. doi: 10.3390/cancers13174359 (PMC8430832; doi:10.3390/cancers13174359)
Supplement: Supplementary file 1 [file cancers-13-04359-s001.zip › cancers-1340089-supplementary.pdf]

Supplementary Material

Western Blot analysis of E-cadherin and Tubulin. Original blot from Figure 3A and corresponding densitometry readings. Antibodies are indicated in green. Molecular weight markers are labeled in blue and red.

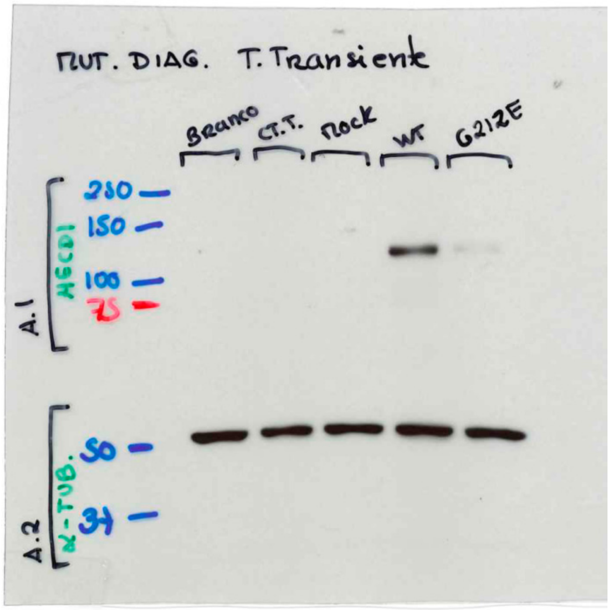

**Band**	**E-cadherin density (INT/mm2)**	**E-cadherin without background**	**Tubulin density (INT/mm2)**	**Tubulin without background**	**E-cadherin / Tubulin**
Parental	5217.51	106.07	10108.78	4736.35	0.02
Mock	5249.96	138.51	10275.98	4903.55	0.02
WT	10317.05	5205.61	10598.41	5225.98	0.99
G212E	5893.10	781.65	10223.34	4850.91	0.16
Background	5111.44	0.00	5372.43	0.00	0.00
